# Sec62 promotes early recurrence of hepatocellular carcinoma through activating integrinα/CAV1 signalling

**DOI:** 10.1038/s41389-019-0183-6

**Published:** 2019-12-10

**Authors:** Juan Du, Zhihao Zhao, Hetong Zhao, Dong Liu, Hui Liu, Jun Chen, Binbin Cheng, Xiaofeng Zhai, Zifei Yin, Yani Zhang, Changquan Ling

**Affiliations:** 1 Department of Traditional Chinese Medicine, Changhai Hospital, Navy Military Medical University, Shanghai, 200433 China; 2 The Third Department of Hepatic Surgery, Eastern Hepatobiliary Surgery Hospital, Navy Military Medical University, Shanghai, 200433 China; 3 Liver Cancer Institute, Zhongshan Hospital, Fudan University, Shanghai, 200433 China

**Keywords:** Tumour biomarkers, Metastasis, Tumour biomarkers, Metastasis

## Abstract

Postsurgical recurrence within 2 years is the major cause of poor survival of hepatocellular carcinoma (HCC) patients. However, the molecular mechanism underlying HCC recurrence remains unclear. Here, we distinguish the function and mechanism of Sec62 in promoting HCC recurrence. The correlation between Sec62 and early recurrence was demonstrated in 60 HCC samples from a prospective study. HCC cells with Sec62 knockdown (Sec62^*KD*^) or overexpression (Sec62^*OE*^) were used to determine the potential of Sec62 in cell migration in vitro. Microarray analysis comparing Sec62^*KD*^ or Sec62^*OE*^ to their control counterparts was used to explore the mechanisms of Sec62-induced recurrence. A luciferase-labelled orthotopic nude mouse model of HCC with Sec62^*KD*^ or Sec62^*OE*^ was used to validate the potential of Sec62 in early HCC recurrence in vivo. We found that high expression of Sec62 was positively correlated with surgical recurrence in clinical HCC samples. Multivariate analysis revealed that Sec62 was an independent prognostic factor for early recurrence in postoperative HCC patients. Moreover, Sec62 promoted migration and invasion of HCC cells in vitro and postsurgical recurrence in vivo. Mechanically, integrinα/CAV1 signalling was identified as one of the targets of Sec62 in cell movement. Overexpression of integrin α partially rescued the Sec62 knockdown-induced inhibition of cell migration. Sec62 is a potentially prognostic factor for early recurrence in postoperative HCC patients and promotes HCC metastasis through integrinα/CAV1 signalling. Sec62 might be an attractive drug target for combating HCC postsurgical recurrence.

## Introduction

Hepatocellular carcinoma (HCC) was the fourth leading cause of cancer death worldwide in 2018, with ~841,000 new cases and 782,000 deaths annually^1^. The major risk factors of HCC vary from region to region. In highest incidence areas of HCC (China, Eastern Africa), the key determinants are chronic hepatitis B virus (HBV) infection and aflatoxin exposure, whereas in other countries (Japan, Egypt), HCV infection is likely the predominant cause^2^. Notably, the mortality rates of HCC have increased in northern and central Europe and the USA, where the leading risk factors include alcohol consumption, increased overweight/obesity, and diabetes^3^. Surgical resection, liver transplantation or local ablation are recommended for patients with very early stage tumours (Barcelona Clinic Liver Cancer [BCLC] 0/A). Liver transplantation is difficult to perform due to its high cost and the scarcity of donor organs in Asian–Pacific countries^4^. For patients with well-preserved hepatic function, surgical resection provides a better clinical outcome than local ablation^5,6^. However, tumour recurrence commonly occurs within two years of resection (19% to 25% in the first year) and exceeds 70% at 5 years^7,8^. Metastasis is the major reason for the high recurrence and poor survival of HCC patients^9,10^. Therefore, it is important to elucidate the molecular mechanisms of HCC metastasis.

Mammalian Sec62 is associated with the Sec61-complex, the main pore for protein translocation in the ER membrane, and with Sec63, an ER membrane protein containing a luminal J-domain^11^. Mammalian Sec62 also interacts with ribosomes via two conserved peptide motifs in its N-terminal cytosolic domain^12,13^. Through these functional elements, Sec62 regulates translation, influences protein translocation to the ER, and indirectly interacts with the key regulator of the unfolded protein response (UPR), BiP (Grp78, HspA5), thereby affecting cellular responses to ER stress inducers. Sec62 was previously characterised as a probable target gene in prostate cancer, lung cancer and thyroid carcinoma due to its high positive rate. By silencing SEC62, cell migration and the invasive potential of prostate cancer cells are markedly reduced, while cell viability is minimally affected^14^. To date, no studies have reported the clinicopathologic significance and potential function of Sec62 in HCC.

Integrins are a group of heterodimeric adhesion receptors that mediate the attachment of cells to extracellular matrices and other cells. Some aspects of integrin function are dependent on the interactions of integrin with its neighbours in the cell membrane and inside the cell. Caveolin, which is a src kinase substrate, is a 210 amino acid (~21 kDa) membrane protein that binds cholesterol and a number of signalling molecules potentially linked with integrin function. Integrin plays a role in migration and tumour metastasis of tumour cells^15^. The integrin αV, 5 and 11 subunits have been reported to be involved in the process of HCC cell migration^16–20^. Moreover, targets of integrins, MLCK and calpain, are required for the invasive and metastatic potential of tumour cells^21–23^.

In this study, we demonstrate that high expression of Sec62 is associated with surgical recurrence of HCC. Sec62 promotes HCC cell migration and invasion by up-regulating integrin α/CAV1 expression. Sec62 might be a prognostic marker and an attractive drug target for combating the postsurgical recurrence of HCC.

## Results

### High expression of Sec62 promotes early recurrence of HCC after curative resection in patients

**Fig. 1:**
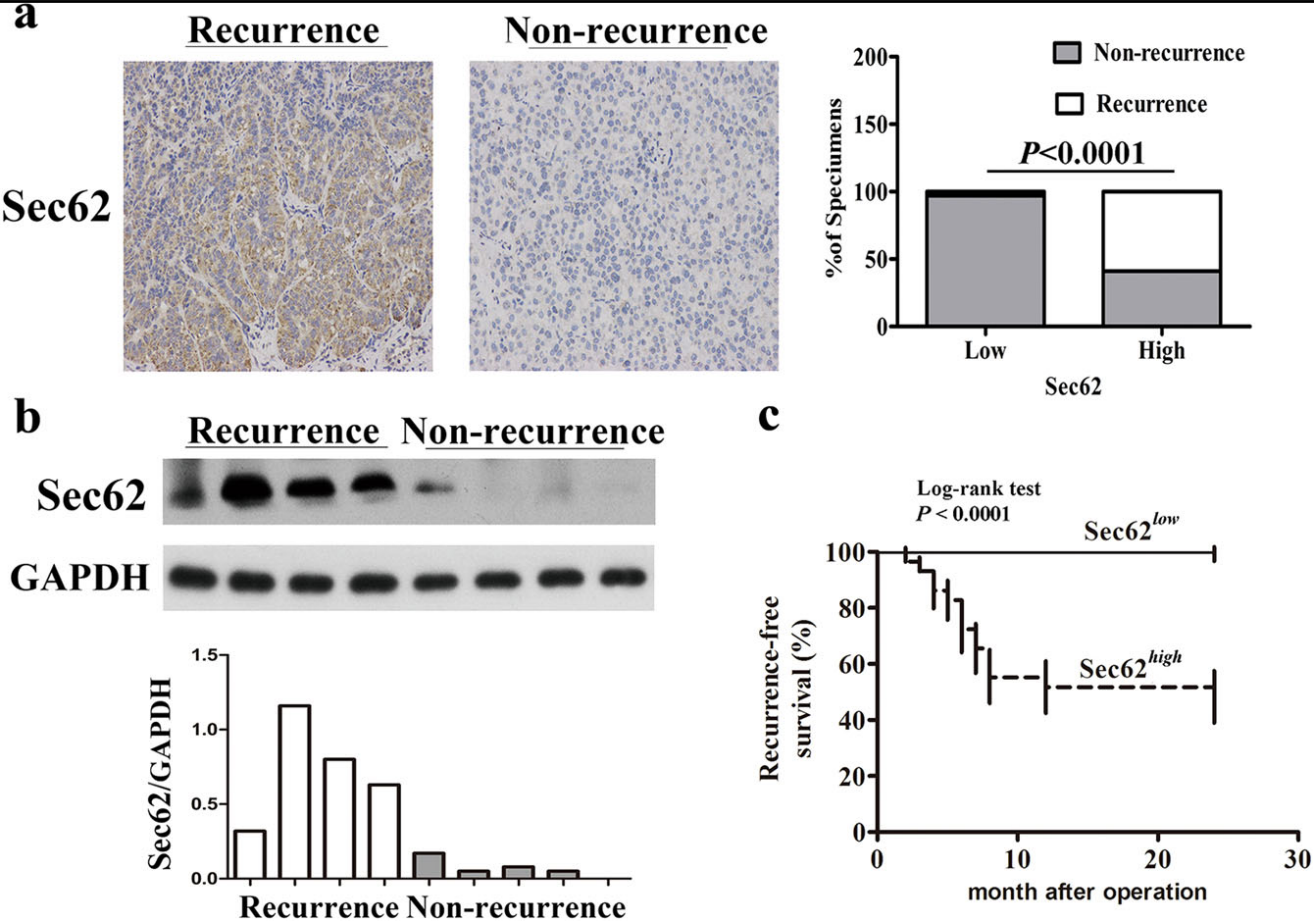
High expression of Sec62 promotes early recurrence HCC after curative resection in patients. **a** Left: Representative immunohistochemical (IHC) staining for Sec62 in primary HCC tissues with early recurrence and non-recurrence (×200). Right: Percentage of samples showing low (*n* = 35) or high Sec62 (*n* = 25) expression in primary HCC with non-recurrence and early recurrence. The *P* values were determined by the Chi-square or Fisher’s exact tests. **b** Western blot analysis of Sec62 in HCC patient samples with recurrence (*n* = 4) or without recurrence (*n* = 4). **c** Kaplan–Meier curves for recurrence-free survival of 60 HCC patients according to high and low Sec62 expression determined by the IHC staining index.

The function and clinical relevance of Sec62 have been poorly described in human HCC. In this study, we evaluated the immunohistochemical expression of Sec62 in tumour tissues from a prospective cohort of 60 HCC patients. After 2 years of follow-up, the levels of Sec62 were much higher in tumour tissues with early recurrence (20/60) than in non-recurrent tumours (40/60) (Fig. 1a left and Fig. 1Sa). We further analysed the correlation between Sec62 expression and early HCC recurrence. The expression levels of Sec62 were positively correlated with early HCC recurrence (Fig. 1a right). Meanwhile, Western blot analysis showed that the Sec62 levels in tumour tissues with early recurrence were much higher than those from non-recurrent tumours (Fig. 1b). Furthermore, higher expression of Sec62 was associated with shorter recurrence-free survival of patients. Multivariate analysis revealed that Sec62 expression was an independent and significant risk factor for HCC recurrence (Fig. 1c). The area under the ROC curve (AUC) was 0.89 (95% CI 0.81–0.98) (Fig. 1Sb). These results suggest that Sec62 is closely correlated with early recurrence and may be a potential predictor for postsurgical recurrence of HCC.

### Sec62 promotes HCC cell migration and increases its invasive potential

**Fig. 2:**
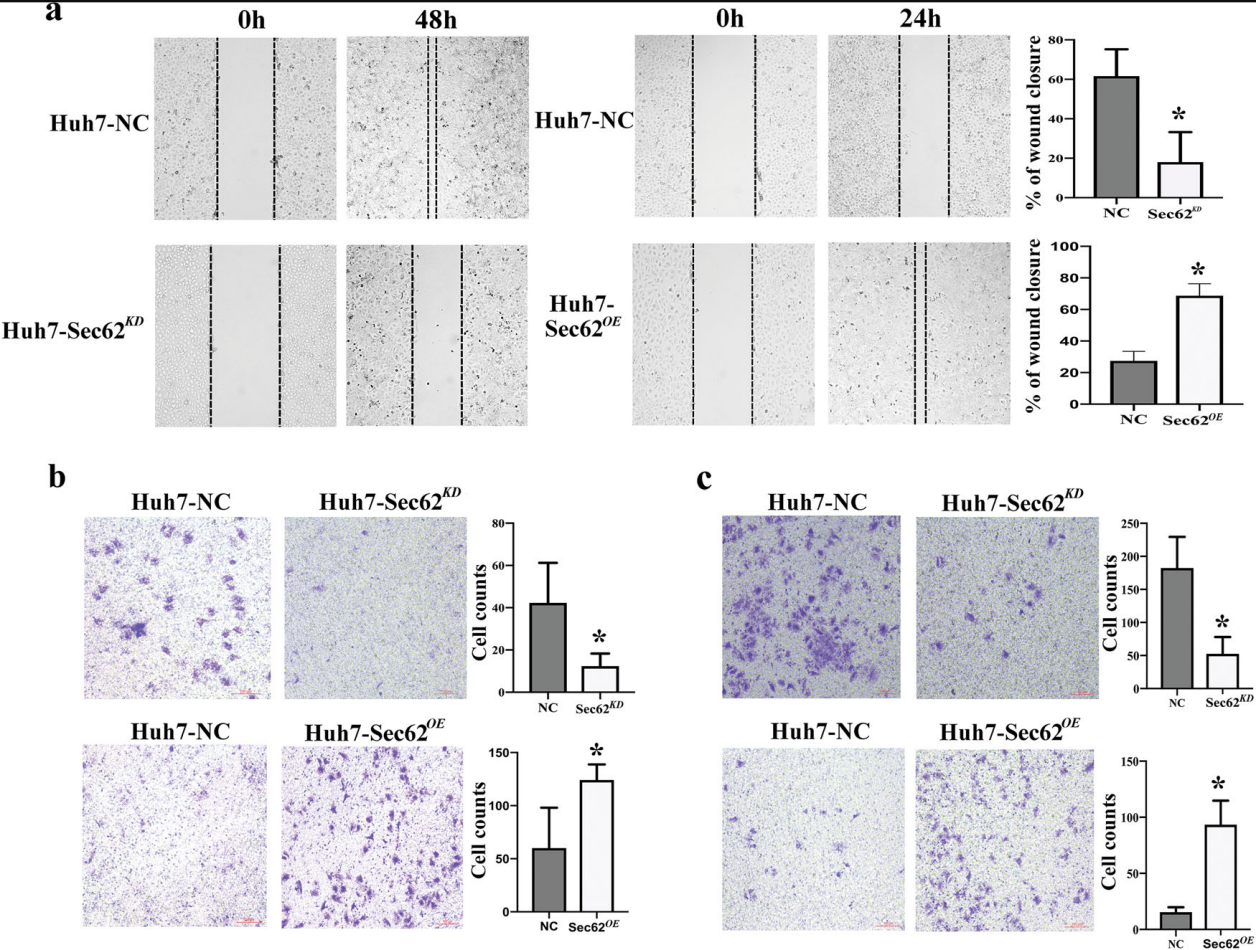
Effects of Sec62 on the migration and invasive potential of Huh7 HCC cells. Huh7 cells were transfected with LV- Sec62 shRNA, LV-Sec62 or LV-mock. Then, stable clones were selected. **a** The motility of these cells was evaluated by a monolayer wounding assay. Transwell assay analysis of the migration (**b**) and invasion (**c**) abilities of the indicated cells. The number of cells that invaded through the filter into the lower compartment was determined using a colorimetric crystal violet assay. Data are presented as the means ± SD of at least three independent experiments and were compared to the amounts of invaded cells from negative control transfected cells. *P < 0.01 versus the negative control (NC) group (Student’s *t* test).

The protein and mRNA levels of Sec62 in Huh7, Hep G2 and 97H cells were higher than those in the Hep3B, MHCC97-L, and LM3 cell lines as well as human normal liver cells, L02 (Fig. 2Sa). To determine the role of Sec62 in HCC cells in vitro, we first determined whether Sec62 had any effect on HCC cell proliferation. Two stable cell lines, Huh7-Sec62^*KD*^ and Huh7-Sec62^*OE*^, were established. The MTT assay showed that down-regulation or up-regulation of Sec62 had no effect on Huh7 cell proliferation at 24 h (Fig. 2Sb).

**Fig. 3:**
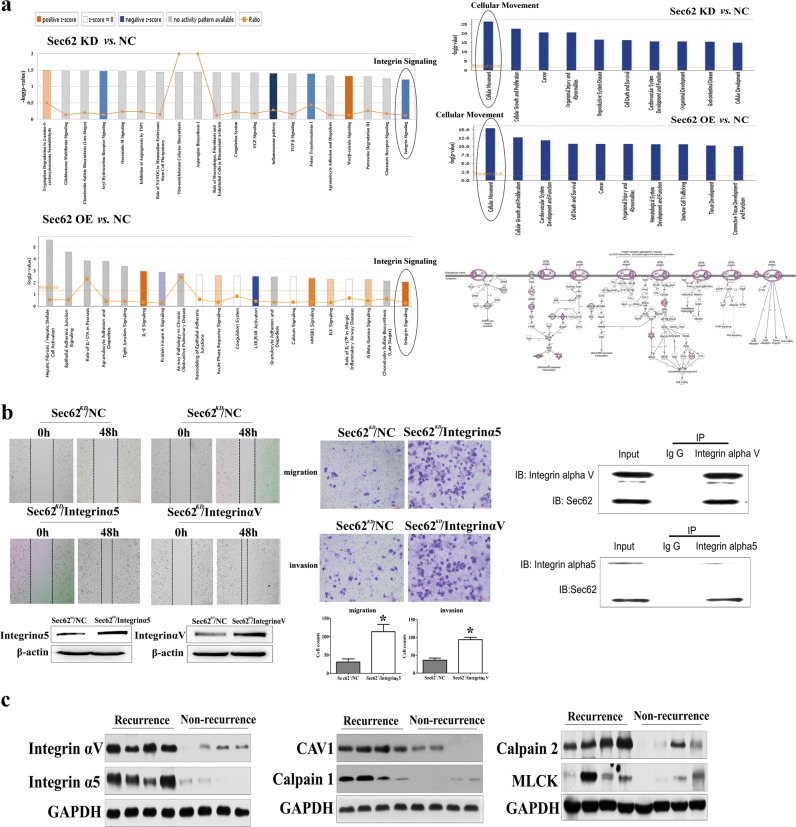
Sec62 regulates cellular movement by targeting integrin α signalling. **a** Left: Functional pathway analysis of the differentially expressed genes was conducted using commercially available IPA software. The change in integrin signalling in both Huh7-Sec62-shRNA cells and Huh7-Sec62-overexpressing cells is consistent with the expression of Sec62. The colour indicates the degree of down- (blue) or up- (orange) regulation following Sec62 knockdown or overexpression in Huh7 cells. Upper right: Cellular movement regulated by Sec62 was identified in both Huh7-Sec62-RNAi cells and Huh7-Sec62-overexpressing cells by GO functional analysis. Lower right: Interaction of Sec62 and integrin signalling after Sec 62 knockdown in Huh7 cells, which was determined based on the integrin signalling interactome in the Ingenuity IPA database overlaid with microarray data from Huh7-Sec62-RNAi cells. **b** Left: Integrin α5 and integrin αV were overexpressed in Sec62-knockdown (Sec62^−^) Huh 7 cells, and the healing ability of these cells was evaluated by a monolayer wounding assay. Western blot analysis of integrin α 5 and integrin αV expression in Sec62-knockdown cells. Middle: Transwell assay analysis of the migration ability of Sec62^−^/ integrin α5 (top) or Sec62^−^/ integrin αV (low) cells. The number of cells that invaded through the filter into the lower compartment was determined using a colorimetric crystal violet assay. Data are presented as the means ± SD of at least three independent experiments and were compared to the amounts of invaded cells from negative control transfected cells. *P < 0.01 versus the control group (Student’s *t* test). Right: Western blot of co-immunoprecipitated integrin αV (top) and integrin α5 (low) in Huh7 cells after Co-IP with an anti-Sec62 antibody. **c** The levels of integrin αV, integrin α 5, CAV1, calpains and MLCK expression in the integrin α/CAV1 pathway from HCC patient samples with recurrence (*n* = 4) or without recurrence (*n* = 4) were evaluated by Western blot analysis.

Cell migration and invasion are characteristics of most malignant tumours. Next, we determined the impact of Sec62 on cell migration and invasion. As shown in Fig. 2a–c, down-regulation of Sec62 decreased the migration and invasion of Huh7 cells, whereas up-regulation of Sec62 expression significantly increased the migration and invasion of Huh7 cells. Using 97H and 97L cells, similar results were observed, as shown in Fig. 3Sa–c: down-regulation of Sec62 decreased the migration and invasion of 97H cells. Up-regulation of Sec62 expression increased the migration and invasion of 97L cells. These results suggest that Sec62 may promote postsurgical recurrence of HCC by increasing HCC cell migration and invasion.

### Sec62 promotes cellular movement via targeting integrin α/CAV1 signalling

**Fig. 4:**
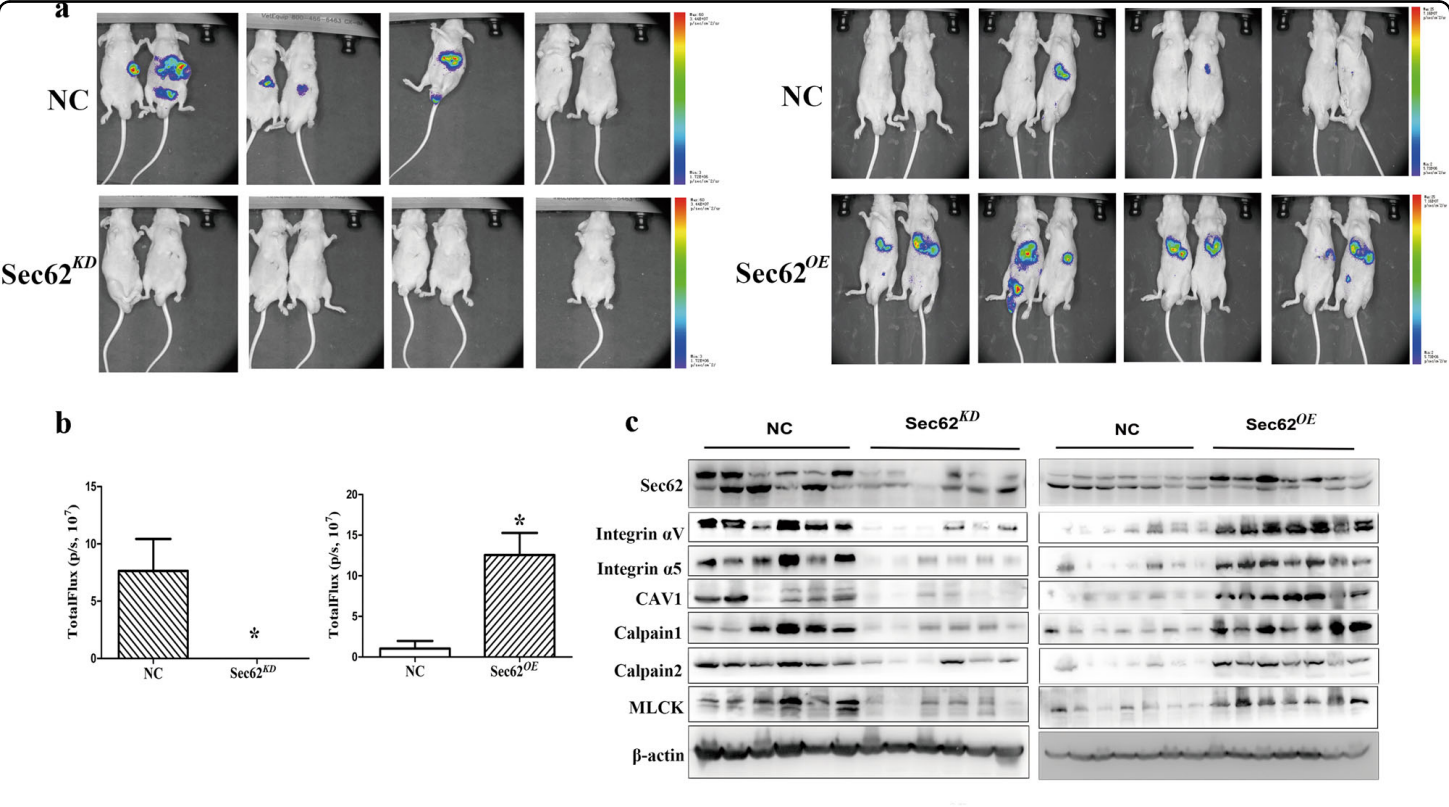
Potential of Sec62 for postsurgical recurrence in an orthotopic xenograft mouse model of HCC. Luciferase-labelled Huh7 cells with or without stable Sec62 knockdown were subcutaneously injected into the right axillary, and then, the xenografts were orthotopically implanted into the livers of nude mice. Mice underwent HCC resection on day 14 after implantation. **a** Left: tumour recurrences were detected after tumour resection with the IVIS system. Then, luciferase-labelled Huh7 cells with or without stable Sec62 overexpression were subcutaneously injected into the right axillary, and then, the xenografts were orthotopically implanted into the livers of nude mice. Mice underwent HCC resection on day 10 after implantation. **a** Right: tumour recurrences were detected after tumour resection with the IVIS system. **b** Quantitative fluorescence data in mice. *P < 0.01 versus the control group (Student’s *t* test). **c** Western blot analysis of Sec62, integrin αV, integrin α 5, CAV1, calpains and MLCK expression in the resected tumour is shown.

To elucidate the molecular mechanisms by which Sec62 contributes to the migration and invasion of HCCs, we carried out microarray analyses comparing the gene expression of Huh7Lv-shSec62 versus Huh7Lv-NS cells and Huh7Lv-Sec62 versus Huh7Lv-NS cells. Gene expression profiling using the Affymetrix GeneChip PrimeView Human Gene Expression Array identified 331 up-regulated and 534 down-regulated transcripts and 146 up-regulated and 45 down-regulated transcripts, which were significantly differentially expressed based on the |FC| > 1.5 and P < 0.01 thresholds, in Huh7 cells with Sec62 knockdown and Sec62 overexpression, respectively, compared with their controls. Moreover, functional gene analysis using Ingenuity Pathway Analysis (IPA) revealed that Sec62 knockdown modulated key pathways typically activated in IL-6 signalling, PPAR signalling, integrin signalling, PI3K/AKT signalling and phospholipase C signalling, while Sec62 overexpression modulated key pathways typically activated in IL-8 signalling, integrin signalling and phospholipase C signalling (|Z-score| > 2, Fig. 3a left). Notably, the integrin pathway and phospholipase C pathway were the common putative signalling pathways identified by Sec62 knockdown and overexpression. Next, ten functional classifications, as annotated by Gene Ontology (GO), were significantly enriched, including cell movement, cellular growth and proliferation, cancer, etc. Cell movement was the most modulated function following both Sec62 knockdown and overexpression (Fig. 3a upper right). Based on the *p*-value and the Gene Ontology analysis, integrin signalling was selected for further analysis. The knowledge-based interactome of Sec62-regulated integrin in Huh7-Sec62 knockdown and Huh7-Sec62 overexpression cells was constructed using IPA and is shown in Fig. 4Sa. To further identify the pathway regulated by Sec62, we examined the knowledge-based integrin pathway using IPA overlaid with microarray data from Huh7-Sec62 knockdown cells (Fig. 3a lower right). Several members of the integrin pathway regulated by Sec62 were confirmed by qRT-PCR and western blot analysis. Among them, the expression of integrin α2b, integrin α4, integrin α5, integrin αV, CAV1, calpain, and MLCK are consistent with microarray data from both qRT-PCR and Western blot analysis (Fig. 4Sb, c).

To validate the role of integrin α in Sec62-induced HCC cell migration and invasion, we overexpressed integrin α2b, integrin α4, integrin α5, and integrin αV in Sec62-knockdown Huh 7 cells and determined their effects on cell migration and invasion. As shown in Fig. 3b and 5S, down-regulation of Sec62 decreased the migration of Huh7 cells, whereas ectopic expression of integrin α5 and integrin αV significantly abrogated Sec62 knockdown-induced inhibition of cell migration. Ectopic expression of integrin α2β, α4, α5 and integrin αV were validated by Western blotting (Fig. 3b and 5S). To further explore the targets of Sec62 that contribute to the corresponding changes in integrin α signalling, co-IP was carried out in Huh7 cells. The results demonstrated that integrin αV or integrin α5 were immunoprecipitated and Sec62 was co-immunoprecipitated together with integrin αV or integrin α5 (Fig. 3b right). Coincidentally, the levels of integrin αV and integrin α5 as well as the integrin α targets CAV1, calpain, and MLCK expression in HCC samples with early recurrence were higher than those in non-recurrence samples (Fig. 3c). These results further indicate that Sec62-induced cell migration is dependent on integrin αV and integrin α5.

### Sec62 is involved in postsurgical recurrence of HCC in the orthotopic xenograft mouse model of HCC

To further investigate the function of Sec62 in early HCC recurrence, we generated mice bearing orthotopically implanted tumours formed from luciferase-labelled Lv-Sec62^*KD*^, Lv-Sec62^*OE*^, or Lv-NC Huh7 cells. Within 14 days after surgical resection, five out of seven mice bearing Lv-NC cells had relapsed with metastasis. In marked contrast, no recurrence was observed in mice bearing Lv-Sec62^*KD*^ cells (Fig. 4a and b left). While 100% of mice bearing Lv-Sec62^*OE*^ cells relapsed within 10 days after surgical resection, but only a fraction of mice (2/7) bearing Lv-NC cells relapsed (Fig. 4a and b right). Western blot analysis showed that Sec62 expression and Sec62 targets integrin α/CAV1 expression in tumour tissues from the surgical resection in the Sec62^*KD*^group were much lower than that in the NC group (Fig. 4c left), while Sec62 expression and integrin α/CAV1 expression in the Sec62^*OE*^ group was much higher than that in the NC group (Fig. 4c right). Collectively, these results suggest that high expression of Sec62 promotes postsurgical recurrence of HCC in an orthotopic xenograft mouse model.

## Discussion

HCC recurrence is a major postoperative complication. In particular, early recurrence (i.e., within 2 years of resection) accounts for more than 70% of tumour recurrence^24^. Identification of patients who are at high risk of recurrence after potentially curative surgery allows clinicians to detect recurrent HCC at its earliest stage, when curative therapy may still be feasible. Potential biomarkers for identification and the mechanisms underlying rapid recurrence need to be explored. Here, we demonstrate the strong promotion potential of Sec62 for HCC early recurrence through activating integrin α/CAV1 signalling. Sec62 might be a prognostic biomarker and an attractive therapeutic target for combating postoperative HCC recurrence.

In recent years, numerous studies have reported the Sec62’s relevance, as a prognostic biomarker to head and neck cancer^25,26^, prostate cancer^27^, and lung cancer^22,25^, indicating a worse outcome for patients with higher Sec62 levels. On the contrary, another cohort of prostate cancer patients with an increased SEC62 level showed a lower risk of recurrence and progression compared with patients without an increased SEC62 level, which might be due to the comparably few patients in this study (*n* = 22)^28^. In the present prospective study, we found that HCC patients (BCLC A) with high expression of Sec62 had higher recurrence rates than patients with low expression of Sec62 within 2 years after surgical resection. By multivariate analysis, Sec62 was an independent and significant risk factor for HCC recurrence. These clinical data are consistent with previous research and strongly suggest that Sec62 plays a role in the early surgical relapse of HCC. To further validate the potential role of Sec62 as a prognostic marker and to establish a valid basis for clinical applications, tumours from more surgical HCC patients need to be studied in the future.

According to our in vitro study, the levels of Sec62 in Huh7, Hep G2 and 97H cells were higher than those in other cell lines. To explore the roles of Sec62 in HCC cell biology, Sec62 knockdown and overexpression in Huh7, 97H and 97L cells were investigated. Our results clearly showed that knockdown or overexpression of Sec62 had no significant effect on Huh7 cell proliferation, consistent with previous reports^14,22,29^. Of note, Sec62 knockdown effectively blocked the migration and invasion of Huh7 cells, whereas exogenous expression of Sec62 notably promoted the migration and invasion of Huh7 cells. Similar results were found in 97H and 97L cells. Consistently, the migration potential of prostate cancer cells, NSCLC cells, thyroid cancer cells, and cervical cancer cells were markedly reduced by Sec62 knockdown, whereas the migration of cervical cancer cells and human embryonic kidney cells were promoted by Sec62 overexpression^22,29^. These effects of Sec62 on cell movement were further confirmed by subsequent GO analysis with a microarray. These results all indicate a positive link between Sec62 and cell migration, which is essential for surgical recurrence and metastasis of tumours.

Although the above studies indicate a specific relevance of Sec62 to HCC cell migration and invasion, the underlying mechanisms remain unclear. As Sec62 takes part in the protein translocation in the ER, it is reasonable to deduce that a distinct subgroup of migration-relevant precursor proteins rely on Sec62 for efficient transport. However, no substrates have been identified up to now. In this study, the integrin α/Cav1 pathway was identified as a pivotal regulator of Sec62-induced cell migration and invasion by microarray analysis. Other Sec62-regulated pathways may also contribute to the recurrence and metastasis of HCC. Integrin α/CAV1 was identified as being down-regulated after Sec62 silencing and up-regulated after Sec62 overexpression. Importantly, integrin α/CAV1-regulated cell movement was the most modulated function following Sec62 knockdown and overexpression according to GO analysis. Integrins/calpain have been reported to be involved in cell migration and tumour metastasis^16,17,22,30–32^. CAV1 is known to be a general regulator of integrin signalling^33^. qRT-PCR and Western blot analysis confirmed that the expression of integrin α2b, integrin α4, integrin α5, integrin αV, CAV1, calpain, and MLCK in the integrin α pathway coincided with the expression of Sec62. However, the levels of SHC and SOS in qRT-PCR were not consistent with those in Western blotting. This phenomenon can be explained by (і) the spatial and temporal intervals between the time and loci of transcription and translation of eukaryotic gene expression and (іі) the different half-life of protein than that of mRNA. As a result, integrins, CAV1, calpain, and MLCK might be major regulators of the integrin pathway in this study. We also found that overexpression of upstream genes of integrin signalling, integrin α5 and integrin αV, partially rescued the decreased migration and invasion of HCC cells induced by Sec62 knockdown; however, integrin α2 and integrin α4 did not have a similar function. These results indicate that Sec62-promoted cell migration and invasion is dependent on integrin α/CAV1 signalling. However, the correlation between Sec62 and integrin α remains unclear. IP data demonstrated that Sec62 could bind to integrin α5 and integrin αV and then regulate integrin α/CAV1 signalling. Importantly, the clinical data strongly suggested that the levels of integrin α5, integrin αV, CAV1, calpain, and MLCK expression, which were positively correlated with Sec62 expression, were higher in HCC early recurrent patients than in non-recurrent patients. Thus, it is reasonable to conclude that integrin α5 and integrin αV are functional targets of Sec62 and play important roles in Sec62-promoted HCC surgical recurrence and metastasis.

Our in vivo data demonstrated that overexpression of Sec62 promoted postsurgical recurrence and metastasis, whereas Sec62 knockdown significantly decreased HCC recurrence after surgery in an orthotopic HCC xenograft nude mouse model. The expression of Sec62 targets, integrin αV, integrin α5, CAV1, calpains, and MLCK, and Sec62 were in substantial agreement in vivo, which is consistent with those of clinical samples. These findings indicate that Sec62 might be a prognostic biomarker and an attractive therapeutic target for combating HCC postsurgical recurrence.

In conclusion, Sec62 promotes HCC relapse and metastasis by targeting integrin α/CAV1 signalling. Thus, targeting Sec62 in patients who have undergone HCC resection might improve their surgical outcome via effective inhibition of tumour relapse and metastasis.

## Materials and methods

### Cell lines and recombinant virus

The liver cancer cell lines Huh7, MHCC97-L and MHCC97-H were purchased from Cell Bank of Type Culture Collection of Chinese Academy of Sciences (Shanghai, China). Lentivirus RNA interference of Sec62 (LV-RNAi Sec62 shRNA, 5′- GTTGCTCGATGCATTCTAT -3′), a negative control lentiviral vector (LV-CON shRNA, 5′-TTCTCCGAACGTGTCACGT-3′), lentiviral packaging plasmids expressing Sec62 (NCBI RefSeq record: NM_003262) and a negative control lentiviral vector were synthesised by GeneChem Co., Ltd. (Shanghai, China). The lentivirus titers were 1 × 10^8^ infectious U/ml. A rabbit anti-human Sec62 antibody (ab 137022) was procured from Abcam. Rabbit anti-integrin α2b (#13807), integrin α4 (#8440), integrin α5 (#98204), integrin αV (#60896), CAV1 (#3267), phospho-SHC (Tyr317, #2431), phospho-SHC (Tyr239/240, #2434), SHC (#2432), SOS1 (#5890), Bcar3 (#24032), Calpain 1 (#2556), Calpain 2 (#2539), antibodies were procured from Cell Signal Technology. Rabbit anti-MLCK (SAB1300116) was procured from Sigma.

### Animals

BALB/c male nude mice (20 ± g, 6 weeks) were housed under standard conditions in Zhongshan hospital. Three of four mice were housed in a cage under 12 h light and 12 h dark. The animal protocols were performed in agreement with the SIBS Guide for the Care and Use of Laboratory Animals and were approved by the Animal Ethics Committee of Zhongshan Hospital, Fudan University.

### Patients

Tumour samples from 60 HCC patients were obtained from Eastern Hepatobiliary Surgery Hospital during 2016–2017 after written informed consent was obtained. All patients had early HCCs with tumour nodules less than 5 cm and underwent hepatectomy after their HCC diagnosis. After their discharge from the hospital, all patients were closely followed up. When intrahepatic recurrence was suspected by volumetric computed tomography and/or magnetic resonance imaging, percutaneous liver biopsy with fine-needle aspiration was performed under ultrasound guidance for histological confirmation of the diagnosis. Table 1S summarises the baseline characteristics of the patient cohort. Patients with missing data were excluded from the analysis.

### Establishment of a Luciferase-Labelled Orthotopic HCC Model

Huh7-Sec62^*KD*^ or Huh7-Sec62^*OE*^ stable transfectants and Huh7-NC cells were established using a lentivirus expressing shSec62 or Sec62 and a scramble control with a luciferase reporter gene as described previously^34^. The cells were subcutaneously inoculated into the right flank of nude mice. Three mice per group were for each kind of cells. Three weeks after injection, we isolated subcutaneous tumours and orthotopically grafted them into the livers of nude mice to establish a luciferase-labelled orthotopic HCC model.

The experiments utilised another 30 nude mice, which were randomly allotted to Huh7-Sec62^*KD*^ (*n* = 7) and NC (*n* = 7), Huh7-Sec62^*OE*^ (*n* = 8) and NC (*n* = 8) sub groups. A left upper abdominal paramedian incision was made under anaesthesia, the left lobe of the liver was exposed and part of the liver surface was mechanically injured with scissors. A piece of the Huh7 tumour ~1 mm in diameter was fixed within the liver tissue, and the abdominal wall was closed. On the 14th day after implantation, the lobes where tumours had been implanted were excised from mice. The tumours were ~3 mm in diameter on the 10th or 14th day. The incisional margin and tumour edge were ±3 mm.

In vivo tumour recurrence was measured with in vivo Imaging System Fx Pro (Carestream Health, USA) according to the location of the luciferase signal during isoflurane anaesthesia in mice. The investigator was blinded to the group allocation during the image exposure period. As anticipated, signals emanating from the liver area indicated intrahepatic recurrence, whereas signals outside the liver area indicated abdominal metastasis.

### Wound-healing assay

Huh7-Sec62^*KD*^ or Huh7-Sec62^*OE*^ cells were implanted into 6-well culture dishes. When the cells grew to 90% confluence, a sterilised tip was used to draw a line with the same width on the bottom of the dishes. Images were captured at 0, 24, 48 h after wounding. The data shown are representative of three independent repeats.

### Migration and invasion assays

Transwell cell migration was quantified by seeding cells (*n* = 5 × 10^4^) in serum-free medium containing 0.1% bovine serum albumin onto the top layer of a 24-well BD BioCoat Matrigel invasion chamber (BD Biosciences) for quantitative assessment of cell invasion. After 24 h, cells were stained and counted on the lower side of the membrane under a light microscope (×200, 10 random fields from each well). All experiments were conducted in quadruplicate.

### Immunohistochemical staining

Immunohistochemical (IHC) was performed on formalin-fixed, paraffin-embedded human tissue sections as previously described^35,36^. Sections were incubated with an anti-Sec62 antibody in a humidified chamber at 4 °C overnight. The following staining was performed using the Envision plus System according to the manufacturer’s instructions. Tissue sections were counterstained with hematoxylin and analysed using light microscopy (Nikon). The staining scores of Sec62 were evaluated by Image-Pro Plus 6.0 based on the integral optical density (IOD). The staining intensity was graded as follows: 1, no staining (IOD = 0); 2, weak staining (IOD < 150); 3, moderate staining (150 ≤ IOD < 1000); and 4, strong staining (IOD ≥ 1000). A score ≤ 2 was defined as low expression of Sec62, and a score > 2 was defined as high expression of Sec62. The outcome assessing was done by another researcher who did not know the follow-up results.

### Co-immunoprecipitation of Sec62

Huh7 cells were lysed in lysis buffer containing 50 mM HEPES, 150 mM NaCl (4.38 g), 1 mM EDTA, 1% (w/v) CHAPS and Sigma protease inhibitor cocktail (with or without 50 μg/ml of RNaseA) at room temperature for 10 min. M2 FLAG agarose resin (40 μl) was prepared as described by the manufacturer’s instructions and incubated with 1000 μl of the cell lysate supernatant with gentle agitation at 4 °C overnight. The IP samples were spun down at 8000 rpm for 30 s and washed with wash buffer (50 mM HEPES, 150 mM NaCl, 0.1% Triton X-100, 10% glycerol, pH to 7.5) three times. Then, the proteins were eluted with FLAG peptide (200 ng/μl) and transferred into a new tube for further analysis.

### Statistical analyses

Statistical analyses were performed using SPSS version 17.0 (SPSS, Chicago, IL). All data were presented as the means ± standard deviation (S.D). The *χ*^2^ test, Fisher’s exact probability test, and Student’s *t* test were used for comparisons between groups. The probability of recurrence-free survival was analysed by the Kaplan–Meier method, and differences between groups were estimated by the log-rank test. *p* < 0.05 (two-tailed) was considered statistically significant. The predictive accuracy was calculated using the ROC. For animal studies, sample size was estimated to be at least seven mice per group to ensure power with statistical confidence Statistical differences between two groups.

## Supplementary information

Table 1S

supplementary table legends

Figure 1S

Figure 2S

Figure 3S

Figure 4S

Figure 5S

Supplementary figure legends

## Ehtical approval

The study was approved by Changhai Hosipital Ethic Committee in Changhai Hospital, Eastern Hepatobiliary Surgery Hospital, Navy Military Medical University and Zhongshan Hospital, Fudan University. The study had been registered with Chinese Clinical Trials Registry, number ChiCTR-TRC-07000033 and was performed in accordance with the Declaration of Helsinki. Animal experiments were approved by Animal Ethics Committee of Zhongshan Hospital. Only male mice were used since the study aimed to identify mechanisms and a prognostic biomarker of HCC recurrence, which is high in male.
