# Supplementary material for: Sec62 promotes early recurrence of hepatocellular carcinoma through activating integrinα/CAV1 signalling
Source: Oncogenesis. 2019 Dec 10;8(12):74. doi: 10.1038/s41389-019-0183-6 (PMC6904485; doi:10.1038/s41389-019-0183-6)
Supplement: Supplementary file 1 — Table 1S [file 41389_2019_183_MOESM1_ESM.docx]

**Table 1S. The baseline characteristics of the patient cohort**

| Variables | | Recurrence | Non-recurrence | *P*-Valure |
| --- | --- | --- | --- | --- |
| Age |  | 52.05±8.81 | 55.05±8.27 | 0.11 |
| Sex | Female | 2/20  18/20 | 8/40  32/40 | 0.33 |
|  | male |  |  |  |
| Serum AFP | ≤20ng/ml | 11  9 | 21  19 | 0.85 |
|  | >20ng/ml |  |  |  |
| Virus infection | HBV | 17  0 | 39  1 | 0.51 |
|  | HCV |  |  |  |
| Cirrrhosis | absent | 12  8 | 25  15 | 0.85 |
|  | present |  |  |  |
| Child-pugh score | Class A | 20  0 | 40  0 | 1 |
|  | Class B |  |  |  |
| Tumor number | single | 17  3 | 39  1 | 0.07 |
|  | multiple |  |  |  |
| Maximal tumor size | ≤5cm | 20  0 | 40  0 | 1 |
|  | >5cm |  |  |  |
| Tumor differentiation | I-II | 1  19 | 5  35 | 0.36 |
|  | III-IV |  |  |  |
| TNM stage | I-II | 20  0 | 40  0 | 1 |
|  | III |  |  |  |
