# Supplementary material for: Sec62 promotes early recurrence of hepatocellular carcinoma through activating integrinα/CAV1 signalling
Source: Oncogenesis. 2019 Dec 10;8(12):74. doi: 10.1038/s41389-019-0183-6 (PMC6904485; doi:10.1038/s41389-019-0183-6)
Supplement: Supplementary file 2 — supplementary table legends [file 41389_2019_183_MOESM2_ESM.docx]

**Table 1S. The baseline characteristics of the patient cohort**

Table summarizing the characteristics of patients during hospitalization period of surgical. In the comparison the baseline characteristics between patients with recurrence and non-recurrence

*p*>0.05, χ^2^ test.
