# Supplementary figures and images for: Sec62 promotes early recurrence of hepatocellular carcinoma through activating integrinα/CAV1 signalling
Source: Oncogenesis. 2019 Dec 10;8(12):74. doi: 10.1038/s41389-019-0183-6 (PMC6904485; doi:10.1038/s41389-019-0183-6)

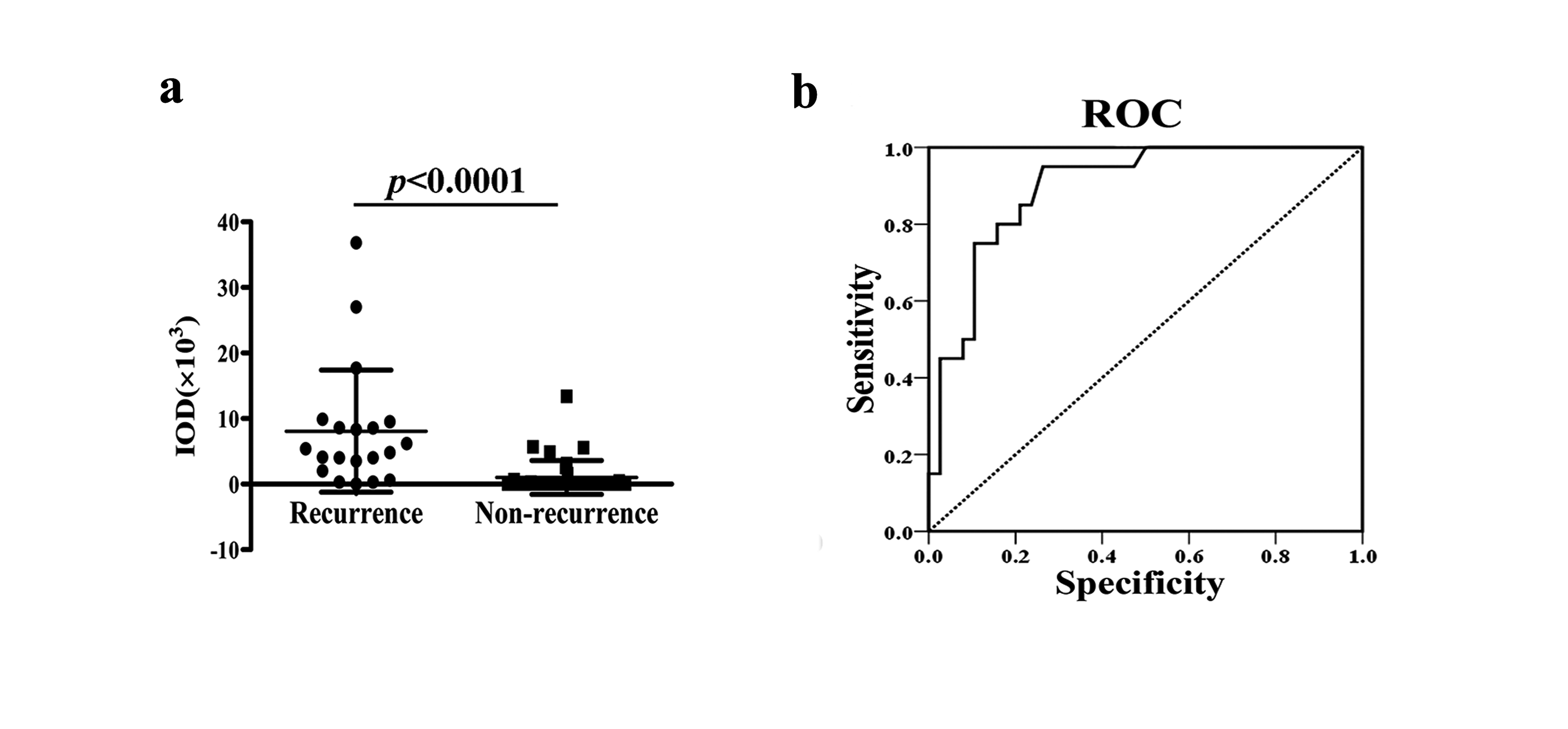

Supplement: Supplementary file 3 — Figure 1S [file 41389_2019_183_MOESM3_ESM.tif]

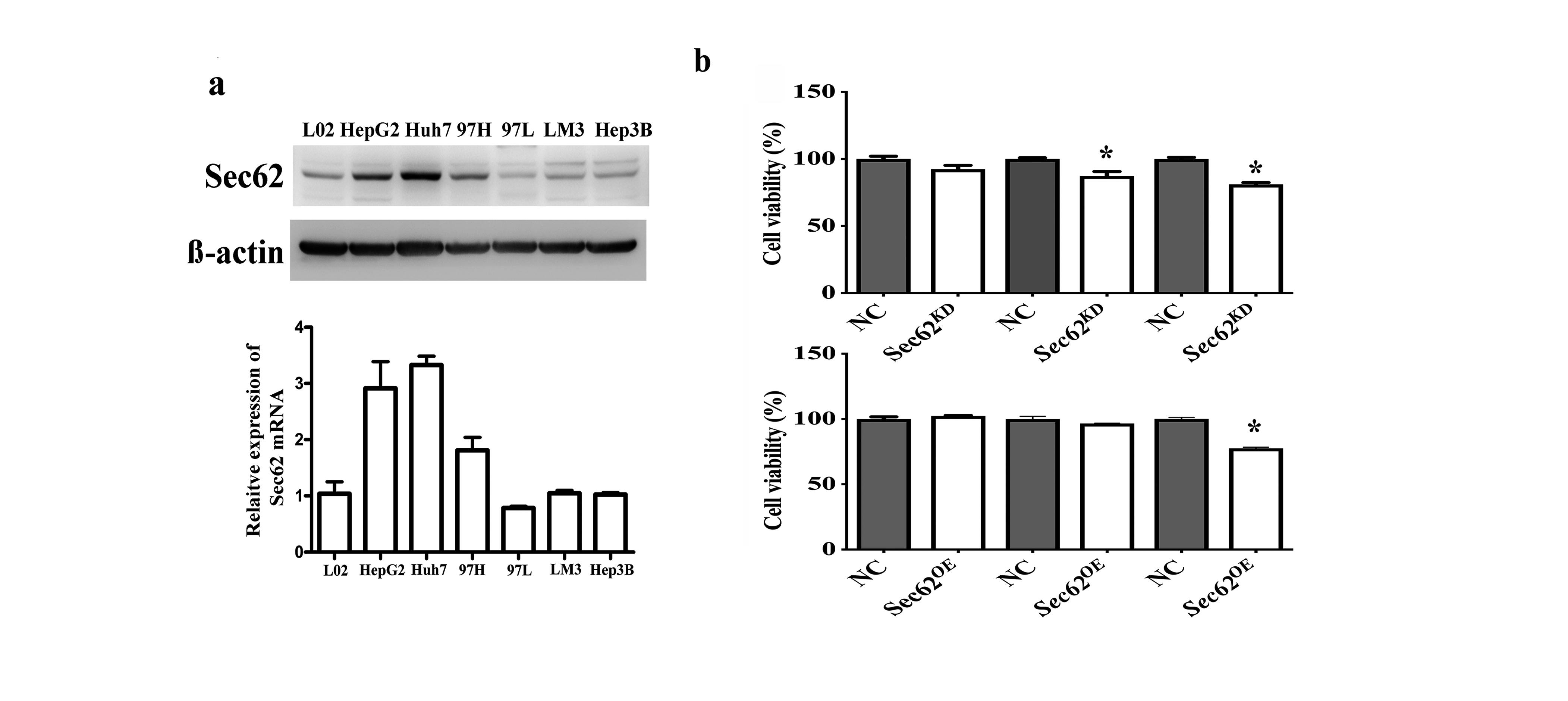

Supplement: Supplementary file 4 — Figure 2S [file 41389_2019_183_MOESM4_ESM.tif]

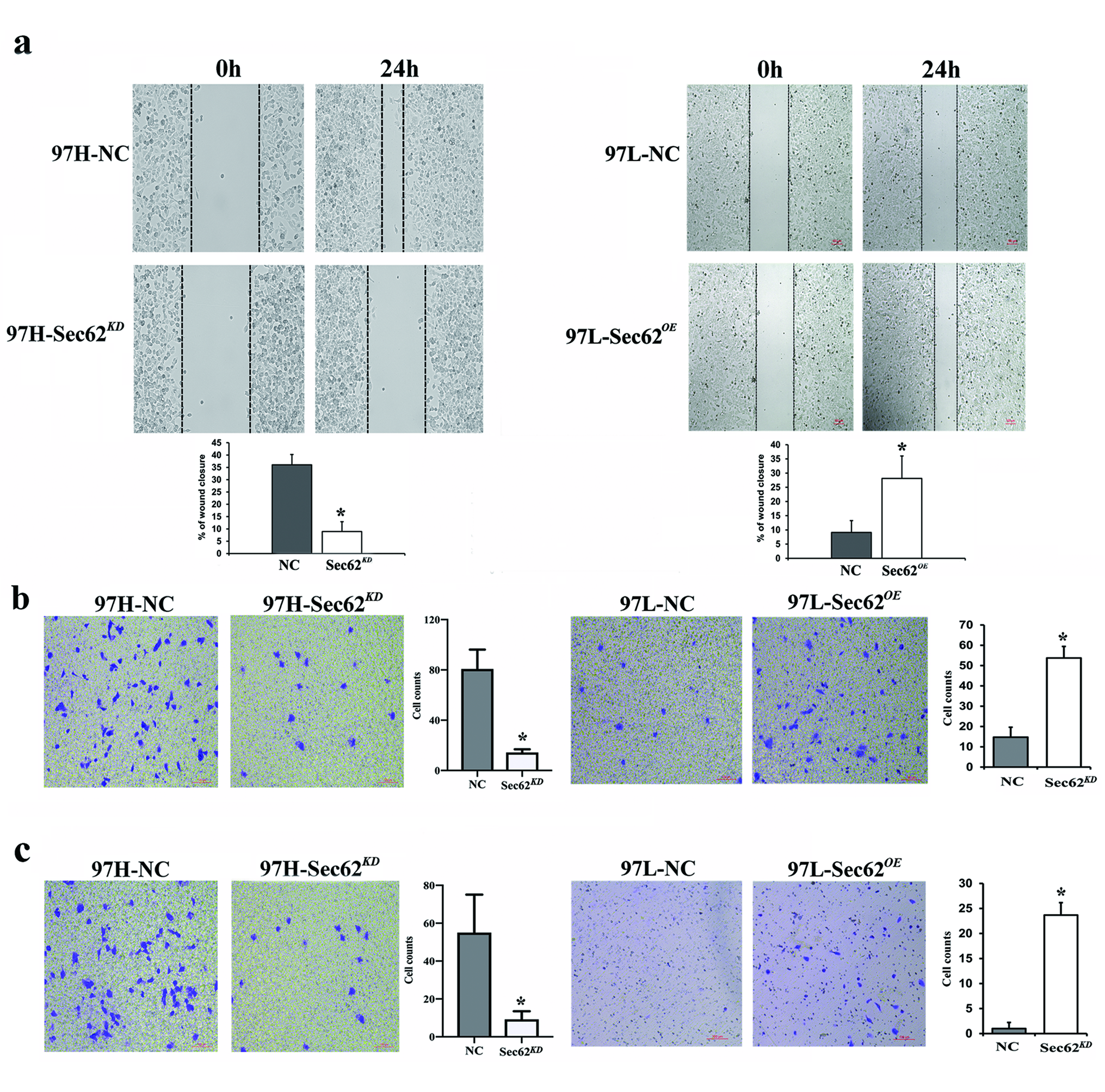

Supplement: Supplementary file 5 — Figure 3S [file 41389_2019_183_MOESM5_ESM.tif]

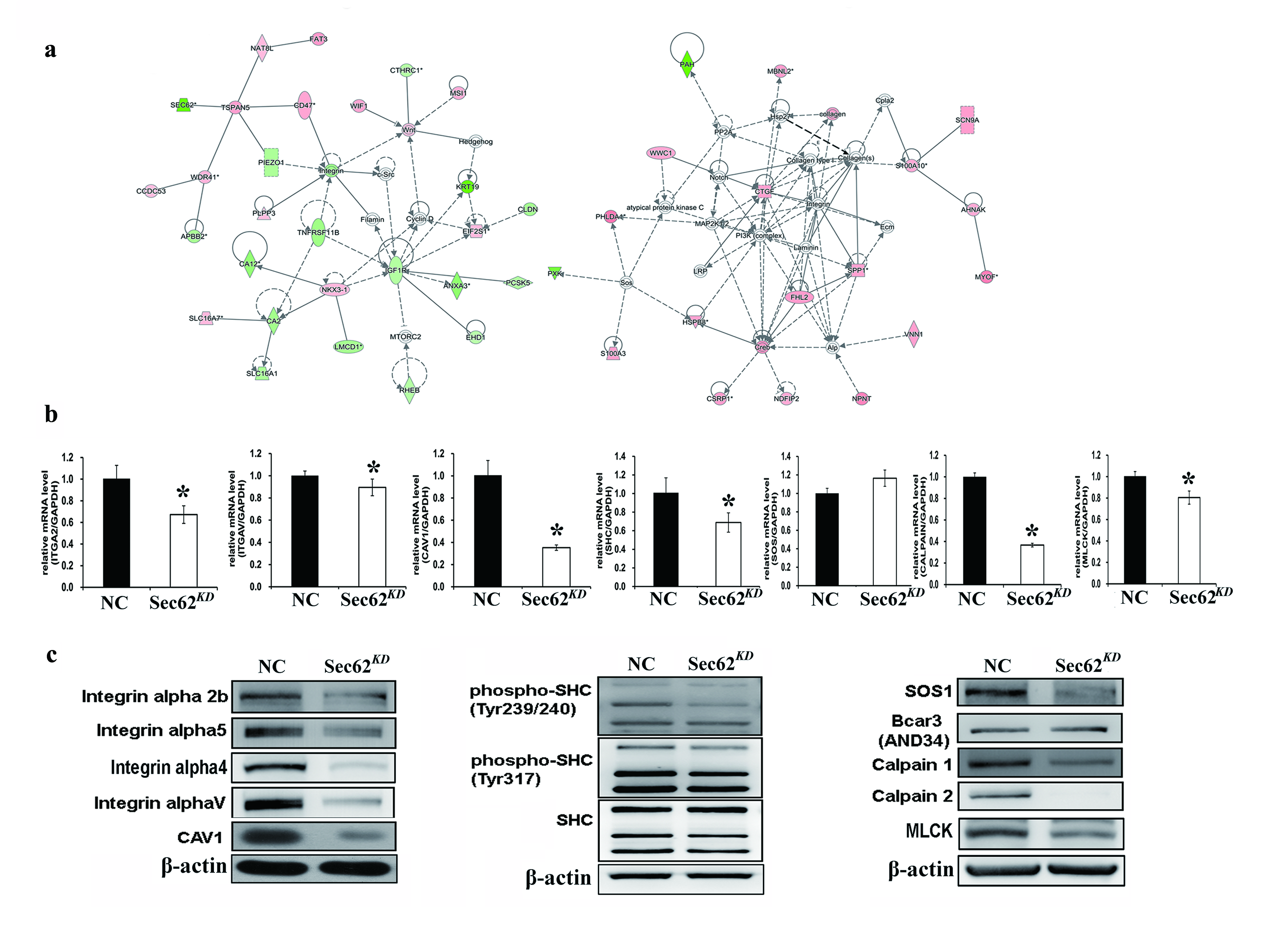

Supplement: Supplementary file 6 — Figure 4S [file 41389_2019_183_MOESM6_ESM.tif]

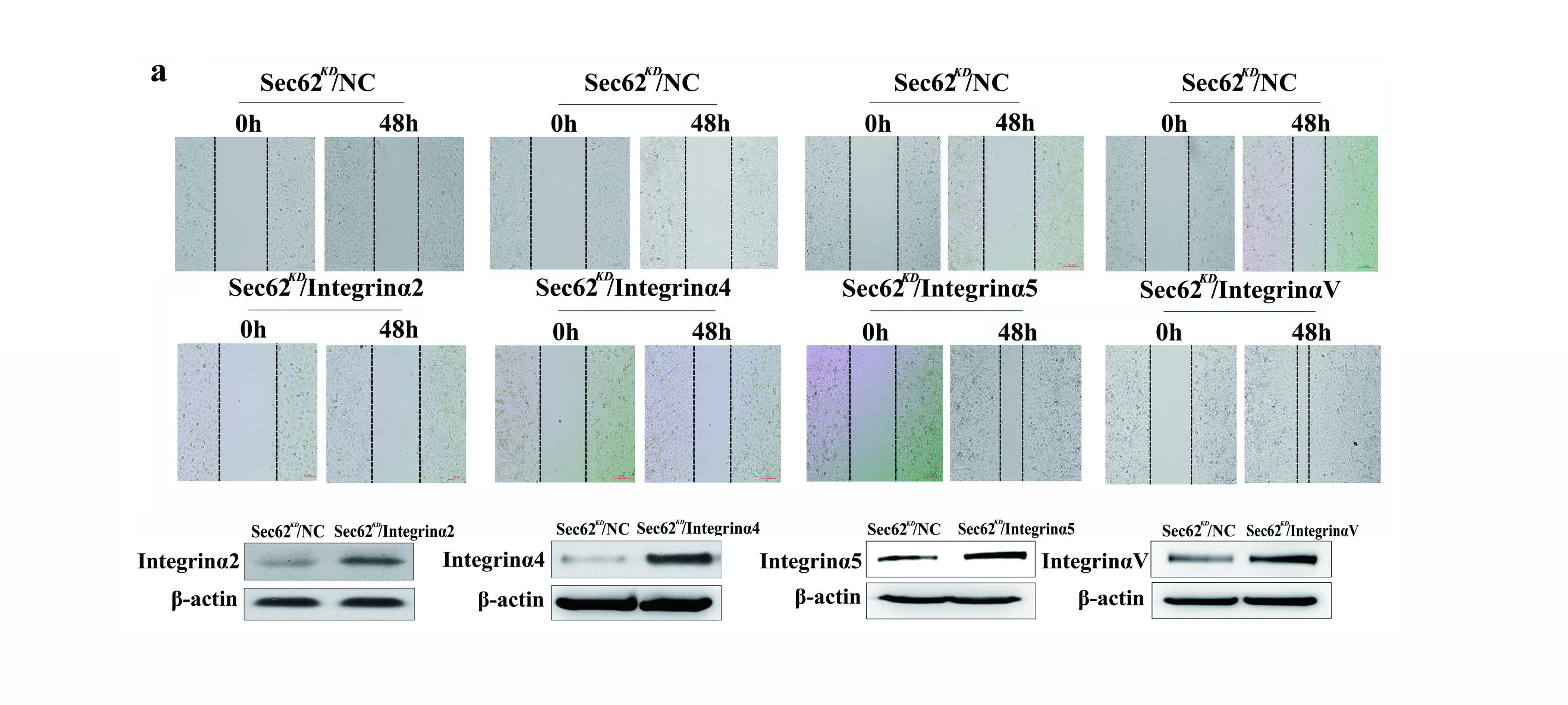

Supplement: Supplementary file 7 — Figure 5S [file 41389_2019_183_MOESM7_ESM.tif]
