# Supplementary material for: Sec62 promotes early recurrence of hepatocellular carcinoma through activating integrinα/CAV1 signalling
Source: Oncogenesis. 2019 Dec 10;8(12):74. doi: 10.1038/s41389-019-0183-6 (PMC6904485; doi:10.1038/s41389-019-0183-6)
Supplement: Supplementary file 8 — Supplementary figure legends [file 41389_2019_183_MOESM8_ESM.docx]

**Supplementary figure legends**

**Fig.1S** **High expression of Sec62 promotes early recurrence HCC after curative resection in patients.** (a) Quantitative data of Sec62 expression in HCC tissues. The *P* values were determined by Student’s t-test. (b) ROC analysis was used to evaluate Sec62 in early HCC recurrence. The AUC was 0.894.

**Fig.2S Effects of Sec62 on Huh7 HCC cells proliferation.** (a) The levels of Sec62 protein (top) and mRNA (low) in different HCC cells. Huh7 cells were transfected with LV- Sec62 shRNA, LV-Sec62 or LV-mock. Then, stable clones were selected. (b) The viability of these cells was evaluated by MTT. The effects of Sec62 knockdown (top) or overexpression (low)on cell viability at 24, 48 and 72 hours. Data are presented as the means±SD of at least three independent experiments and were compared to the amounts of invaded cells from negative control transfected cells. **P* < 0.01 versus the negative control (NC) group (Student’s t-test).

**Fig.3S Effects of Sec62 on migration and invasive potentials of MHCC 97H and 97L cells.** MHCC 97H cells were also transfected with LV- Sec62 shRNA or LV-mock. And MHCC 97L cells were transfected with LV- Sec62 or LV-mock. Then, stable clones were selected. (a) The motility of these cells was evaluated by monolayer wounding assay. Transwell assay analysis of the migration (b) and invasion (c) abilities of the indicated cells. The number of cells that had invaded through the filter into the lower compartment was determined using a colorimetric crystal violet assay. Data are presented as means±SD of at least three independent experiments and were compared to the amounts of invaded cells from negative control transfected cells. **P* < 0.01 versus to the negative control (NC) group (Student’s t-test).

**Fig. 4S Sec62 regulates cellular movement by targeting integrin α signalling.** (a) Knowledge-based interaction network of Sec62 and Sec62 targets after Sec62 knockdown or overexpression in Huh7 cells. (b) Quantification of gene expression in the integrin α/CAV1 pathway of Huh7-Sec62-RNAi cells using real-time PCR. **P* < 0.01 versus the negative control (NC) group (Student’s t-test). (c) Protein expression of the components of the integrin α/CAV1 pathway in Huh7-Sec62-RNAi cells was measured using Western blot analysis.

**Fig. 5S To compare Integrins α function in Sec62-induced migration**

(a) Integrin α2, integrin α4, integrin α5 or integrin αV were overexpressed in Sec62-knockdown (Sec62^-^) Huh 7 cells, and the healing ability of these cells was evaluated by a monolayer wounding assay. Western blot analysis of integrin α2, integrin α4, integrin α 5 or integrin αV expression in Sec62-knockdown cells.
